# Cohort Profile: The Early Childhood Longitudinal Survey (ELPI), Chile

**DOI:** 10.1093/ije/dyag105

**Published:** 2026-07-13

**Authors:** Pablo Martínez, Vania Martínez, Gabriel Sotomayor López, Vicky Rojas, Jenny Encina, Matías Cociña, Massimiliano Orri

**Affiliations:** McGill Group for Suicide Studies, Department of Psychiatry, Douglas Mental Health University Institute, McGill University, Montreal, Québec, Canada; Centro de Estudios en Psicología Clínica y Psicoterapia (CEPPS), Facultad de Psicología, Universidad Diego Portales, Santiago, Chile; Nucleus to Improve the Mental Health of Adolescents and Youths (IMHAY), Santiago, Chile; Millennium Institute for Research in Depression and Personality (MIDAP), Santiago, Chile; Nucleus to Improve the Mental Health of Adolescents and Youths (IMHAY), Santiago, Chile; Millennium Institute for Research in Depression and Personality (MIDAP), Santiago, Chile; Centro de Medicina Reproductiva y Desarrollo Integral del Adolescente (CEMERA), Facultad de Medicina, Universidad de Chile, Santiago, Chile; División de Observatorio Social, Ministerio de Desarrollo Social y Familia de Chile, Santiago, Chile; Escuela de Sociología, Facultad de Ciencias Sociales y Humanidades, Universidad Diego Portales, Santiago, Chile; Dirección de Estudios Sociales, Pontificia Universidad Católica de Chile, Santiago, Chile; División de Observatorio Social, Ministerio de Desarrollo Social y Familia de Chile, Santiago, Chile; Observatorio de Desigualdades, Facultad de Ciencias Sociales y Humanidades, Universidad Diego Portales, Santiago, Chile; McGill Group for Suicide Studies, Department of Psychiatry, Douglas Mental Health University Institute, McGill University, Montreal, Québec, Canada; Department of Epidemiology, Biostatistics, and Occupational Health, School of Population and Global Health, McGill University, Montreal, Quebec, Canada; Danish Research Institute for Suicide Prevention, Mental Health Centre Copenhagen, Copenhagen, Denmark

**Keywords:** early childhood, child development, birth cohort, nationally representative, Chile

Key Features

- The Early Childhood Longitudinal Survey (Encuesta Longitudinal de Primera Infancia, ELPI) was established to provide the first nationally representative longitudinal evidence on early childhood development in Chile and to document how social, familial, and policy contexts shape developmental inequalities from early life into adolescence.
- ELPI is based in Chile and began baseline data collection in 2010. The baseline cohort included 15 175 children born between January 2006 and August 2009, selected using a stratified, two-stage cluster design to ensure national representativeness across urban and rural areas. Children were aged 0–4 years at baseline and represented diverse socioeconomic and geographic backgrounds.
- Participants were followed up in 2012, 2017, and 2024. Attrition reached 34.1% by 2024, leaving approximately two-thirds of the original 2010 cohort currently under follow-up.
- The study collects extensive data on cognitive, socio-emotional, and physical development; family structure and home environment; parenting practices; caregiver mental health; socioeconomic conditions; childcare and education; and health behaviors.
- ELPI data are publicly available through Chile’s Banco Integrado de Datos. Researchers can access de-identified datasets and documentation following standard application procedures, enabling national and international collaboration.

## Why was the cohort set up?

Chile is often considered a success story in Latin America due to its steady economic growth, strong institutions, and the significant reduction of poverty over recent decades (see Table 1 for key country indicators) [1]. According to United Nations Development Program analyses, the national poverty rate dropped markedly between the 1990s and 2022, and access to education, healthcare, and social services has expanded [2]. Despite these advances, Chile remains among the most unequal societies in the region, with persistent gaps in wealth distribution, housing conditions, and educational opportunities [3]. Multiple studies underscore that large portions of the population face precarious labor markets, limited upward mobility, and stark rural-urban disparities [4]. While gross domestic product per capita has increased and extreme poverty has declined, many families still lack consistent access to quality services and face constraints that widen developmental inequities among children [5].

**Table 1.** Key country indicators for Chile

Indicator	Data
Total population	18 480 432 people
Total surface area	2 006 096.3 km^2^
GDP per capita (US$, PPP)	US$ 35 146
Life expectancy at birth	81.1 years (both sexes)
Adult literacy rate	97.8%

Total population, life expectancy at birth, and adult literacy rate were obtained from the 2024 Census results published by the National Institute of Statistics of Chile (INE). Data on GDP per capita were extracted from the annual macroeconomic indicators published by the Central Bank of Chile, based on projections from the International Monetary Fund (IMF) for 2025. Data on total surface area were obtained from the Library of the National Congress of Chile.

Over the past two decades, a few prospective cohort studies have emerged in Chile, mostly following adults or small, region-specific samples. For example, the Limache birth cohort [6], launched in the early 2000s, assessed individuals born between 1974 and 1978 to investigate respiratory health, cardiovascular risk factors, and the long-term effects of early undernutrition. Although pioneering, this cohort focused on a narrow birth window and addressed older cohorts rather than children in contemporary settings [6]. More recently, the Maule Cohort (MAUCO) recruited adults aged 38–74 in a largely rural county to examine cardiovascular diseases, cancer risk, and environmental exposures [7]. While MAUCO addresses pressing health issues and has generated valuable insights into chronic conditions, it begins well after childhood and does not capture the critical processes shaping early developmental outcomes [7]. In parallel, other national longitudinal initiatives, such as the Chilean Longitudinal Social Study (ELSOC) and the Longitudinal Study of Intercultural Relations (ELRI) [8, 9], have advanced understanding of social and cultural processes, and one region-based child cohort has provided valuable early-life data by following children recruited at 12–15 months until preschool entry [10]. However, no fully national, representative child cohort capable of capturing early-life determinants and developmental inequities currently exists.

With this backdrop, the Early Childhood Longitudinal Survey [Encuesta Longitudinal de Primera Infancia (ELPI)] was conceived in 2010 to provide a comprehensive, nationally representative view of young children’s growth and well-being [11]. Policy-makers and researchers recognized that no large-scale resource existed to track cognitive, physical, and socio-emotional development from the earliest years [11]. By capturing a range of factors—such as family environments, childcare arrangements, maternal mental health, and household socioeconomics—ELPI aims to reveal how these conditions shape youth’s developmental trajectories into adolescence. Its broad coverage addresses the need to evaluate large-scale public initiatives (e.g. childcare expansion, parental leave), document the interplay of social context and child outcomes, and guide evidence-based strategies to reduce Chile’s enduring socioeconomic inequalities. Thus, ELPI provides crucial longitudinal data on early childhood development, bridging an important gap between Chile’s adult-focused cohorts and the urgent policy questions regarding children’s formative years [11]. ELPI has been funded by the Chilean State through the responsible ministries (i.e. the Ministry of Education, the Ministry of Labor and Social Welfare, and the Ministry of Social Development and Family) across survey waves.

## Who is in the cohort?

ELPI is an ongoing population-based longitudinal study that follows a nationally representative sample of Chilean children from birth through adolescence. The study started in 2010, and participants were selected using a two-stage stratified cluster design. First, municipalities across Chile (urban and rural) were selected based on region, per capita income, and total population. Second, children born between 1 January 2006 and 31 August 2009, in these municipalities were randomly drawn from the National Civil Registry [11, 12]. This approach ensured coverage of various socioeconomic strata, including remote areas.

Initial invitations targeted approximately 25 000 families, oversampled to achieve a final baseline sample of 15 175 children (i.e. 51% of contacted households participated). Eligible participants had to be permanent residents of Chile, with at least one parent or guardian available for interviews and assessments. Children were excluded if families declined participation or could not be reached. There were no exclusion criteria based on health or family structure, ensuring the inclusion of Chile’s diverse early childhood population. The recruitment team conducted home visits and telephone contact, emphasizing the importance of longitudinal participation. The 2012 and 2017 waves included new cohorts of younger children, whereas the 2024 wave focuses solely on the original 2010 cohort due to operational constraints [11, 12].

All waves of ELPI underwent institutional review by relevant ethics committees in Chile, ensuring compliance with national and international standards on research with human subjects. Written informed consent was obtained from primary caregivers at each wave, with age-appropriate assent collected from older children participating in direct assessments. Data confidentiality is protected under Chilean law; participants’ identifying information is stored separately from their survey responses, and only de-identified datasets are shared with approved researchers. Ethical oversight continues for the 2024 wave, including periodic review of data collection and privacy protocols to guarantee ongoing protection of participants’ rights.

## How often have they been followed up?

In 2010 (Wave 1), 15 175 children aged 0–4 underwent anthropometric measurements, standardized developmental tests, and their caregivers were interviewed face-to-face. The original cohort was re-contacted in 2012 (Wave 2), when a sample of newborns (through 2011) was added. In 2017 (Wave 3), researchers followed up on the 2010 and 2012 samples, plus another sample of children aged 0–5. That wave introduced assessments of executive functions, maternal mental health, and extended socio-emotional and educational outcomes [11]. The 2024 wave (Wave 4) focuses exclusively on the original 2010 children (now adolescents), examining their transitions to secondary education, health behaviors, and future outlook. Logistical constraints prevented the inclusion of a new child cohort and also precluded the follow-up of the 2012 and 2017 cohorts (Table 2) [12].

**Table 2.** Socio-demographic characteristics of ELPI respondents and reference population (CASEN 2009)

Characteristic	ELPI 2010 (*N* = 15 175)	CASEN 2009 (*N* = 11 117)
Maternal age, mean (years)	29.5	29.3
Married/cohabiting (%)	70.9	73.1
Urban residence (%)	90.8	87.4
Employed (%)	43.9	39.3
Education (%)		
Primary or less	16.6	17.3
Secondary	57.6	43.5
Higher	25.8	39.2
Household income (CLP, mean)	531 329	729 990

Estimates are based on analytic samples with complete data for each variable; sample sizes vary across characteristics and between the Early Childhood Longitudinal Survey (ELPI) and the National Socioeconomic Characterization Survey (CASEN). Household income, expressed in Chilean pesos (CLP), was adjusted to 2010 values using the Chilean consumer price index. Percentages are weighted using survey weights.

Attrition was observed across subsequent waves of the original 2010 ELPI Cohort (*n* = 15 175). By 2012, 15.0% of the original sample had been lost to follow-up, increasing to 32.6% by 2017 and 34.1% by 2024 [13]. Main reasons for non-response included household mobility, inability to locate participants, and refusal to continue. Attrition analyses indicated that loss to follow-up was not random: households with more educated caregivers, higher income levels, and those residing in the Metropolitan Region were overrepresented among those lost in later waves [13]. The study implemented improved tracking strategies, such as updated phone registries, to reduce potential selection bias due to differential attrition.

The government of Chile has ongoing plans to follow participants using administrative records, pending legal and technical feasibility reviews.

## What has been measured?

ELPI collects information on child and youth development, caregiver functioning, and home environment through standardized evaluations in multiple domains (Tables 3–5). Cognitive outcomes are assessed using the Peabody Picture Vocabulary Test, which has been administered since 2010 to assess receptive vocabulary. In older cohorts, tasks measuring memory, verbal fluency, and executive functions (such as the Hearts & Flowers task and the Backward Digit Span) have been added. For younger children, the Battelle Developmental Inventory is used to screen broad developmental milestones. Socio-emotional and behavioral measures include the Child Behavior Checklist, which tracks internalizing and externalizing problems. In some waves, additional tools such as the Ages and Stages Questionnaire: Social-Emotional or the Test de Autoestima Escolar (School Self-Esteem Test, a Chilean instrument) have been employed, depending on the child’s age. Physical health measurements include height, weight, and head circumference up until the 2017 wave. In later rounds (especially 2017 and 2024), older children report on diet, physical activity, and any chronic conditions. Family and home environment data come from questionnaires on demographics, socioeconomics, and parenting styles. In addition, observational measures of stimulation, discipline, and family interactions were collected by trained assessors by direct observation using the HOME (Home Observation for Measurement of the Environment) instrument. Maternal or primary caregiver health is captured through depression and stress scales (e.g. the Center for Epidemiologic Studies Depression Scale Revised), and some waves include prenatal exposure questions (e.g. maternal tobacco, alcohol, and illicit drug use during pregnancy, number of prenatal medical visits, and self-reported maternal stress or depression). Educational and community factors include childcare enrollment, academic performance, peer relationships, and neighborhood safety. Interviewers are trained to follow standardized protocols for data collection, which typically occurs in-person. Older children may complete self-administered modules, especially in later waves. After each wave, data underwent cleaning and verification to address any inconsistencies or missing responses before the final datasets became available to the research community.

**Table 3.** Cohort timeline

Cohort	2010	2012	2017	2024
2006–09	1–4 years (*n* = 15 175)	3–5 years (*n* = 12 898)	8–11 years (*n* = 10 230)	14–18 years (*n* = 10 003)
2012		1–2 years (*n* = 3135)	6–7 years (*n* = 2142)	NA
2017			1–5 years (*n* = 4935)	NA

NA, not applicable.

**Table 4.** Instruments used in the Early Childhood Longitudinal Survey (ELPI) to assess child and youth development by age and domain

Domain	Instrument	0–2 years	3–6 years	>7 years	>15 years	Type	ELPI wave
General development	Battelle	X	X			DA	I–III
	TADI	X	X			DA	II
	EEDP	X				DA	I
	TEPSI	X				DA	I
Socio-emotional development	ASQ:SE	X				CR	I–III
	CBCL	X	X	X	X	CR	I–IV
	PHQ-4				X	CSR	IV
	TAE			X		CSR	III
	ECLIS			X	X	CSR	III
	BMSLSS		X	X		CSR	III–IV
Executive function	SDT		X			DA	II
	PTT		X			DA	II–III
	HTKS		X			DA	II
	BDS		X	X		DA	II–III
	H&F		X	X		DA	III
Cognition, language, and achievement	WM			X		DA	III
	TVIP	X	X	X	X	DA	I–IV
Early adversity	ACEs				X	CR	IV
Anthropometric measures	Weight	X	X	X		DA	I–III
	Height	X	X	X		DA	I–III
	HC	X	X			DA	III

Instruments were administered at different waves depending on age and cohort. IQ-related instruments were administered once per cohort.

ACEs, adverse childhood experiences; ASQ: SE, Ages and Stages Questionnaires—Socio-Emotional; BDS, Backward Digit Span; BMSLSS, Brief Multidimensional Students’ Life Satisfaction Scale; CBCL, Child Behavior Checklist; CR, caregiver-reported; CSR, child self-report; DA, direct assessment; ECLIS, School Climate Scale; EEDP, Scale of Psychomotor Development Evaluation; H&F, Hearts and Flowers; HC, head circumference; HTKS, Head-Toes-Knees-Shoulders Task; PHQ-4, Patient Health Questionnaire-4; PTT, Pencil Tapping Task; SDT, Sack Delay Task; TADI, Child Learning and Development Test; TAE, School Self-Esteem Test; TEPSI, Psychomotor Development Test; TVIP, Peabody Picture Vocabulary Test; WM, Woodcock-Muñoz.

**Table 5.** Instruments in the Early Childhood Longitudinal Survey (ELPI) to assess caregiver functioning and home environment

Domain	Instrument	Type	ELPI wave
Cognitive	DS (WAIS)	DA	I–II
	VS (WAIS)	DA	I–II
Socio-emotional	BFI	SR	I–II
	PSI, SF	SR	II–IV
	EPDS	SR	II
	CESDR-10	SR	III–IV
Home environment quality	HOME	OBS	I–IV
Parental components	PSOC	SR	III–IV
Anthropometric measures	Weight	DA	I–IV
	Height	DA	I–III

WAIS subtests were administered once per cohort only. In the first three waves, HOME components included both interviewer observations and direct questions. In Wave 4, an updated HOME-21 version was used, consisting only of caregiver-reported items.

BFI, Big Five Inventory; CESDR-10, Center for Epidemiologic Studies Depression Scale—Revised; DA, Direct assessment; DS (WAIS), Digit Span—Wechsler Adult Intelligence Scale; EPDS, Edinburgh Postnatal Depression Scale; HOME, Home Observation for Measurement of the Environment; OBS, Observation; PSCS, Parenting Sense of Competence Scale; PSI: SF, Parenting Stress Index—Short Form; SR, Self-report; VS (WAIS), Vocabulary—Wechsler Adult Intelligence Scale.

## What has it found?

### Cognitive and language development

By age 2, children from low-income households tend to show lower receptive language scores [14, 15]. Maternal education and maternal cognitive abilities are associated with vocabulary outcomes and general cognitive scores [16, 17], likely through differences in linguistic stimulation at home (e.g. reading, frequent and responsive communication). Access to formal childcare has been linked to improved outcomes, particularly for children from low socioeconomic status families [18, 19]. One analysis found that greater intensity of exposure (i.e. number of hours) was associated with stronger developmental indicators in disadvantaged groups [20].

### Socio-emotional and behavioral outcomes

While overall disparities appear smaller than in cognitive outcomes, differences in maternal education are still associated with behavioral variation [21]. Higher maternal depressive symptoms have been linked to elevated internalizing and externalizing scores in children [22]. Associations between parenting style and child outcomes have also been reported: harsh or inconsistent discipline has been linked to behavioral difficulties, whereas warm and structured parenting is associated with fewer problems [23]. Longitudinal analyses suggest that most children follow low-symptom trajectories, although a subset shows persistently elevated behavioral indicators [24].

### Family structure and caregiving context

Transitions such as the departure of a co-resident grandparent have been associated with reductions in language growth rates [25]. Paternal departure, however, has shown no measurable impact on child outcomes when income is held constant, suggesting effects may be attributable to economic changes rather than absence per se [25]. Parental education remains an important correlate of developmental indicators [17]. Studies examining maternal employment find no consistent associations with adverse outcomes when quality childcare is available [26].

### Health and nutrition

Trends in nutritional status suggest an increasing prevalence of overweight during early childhood [27]. Among toddlers (2–3 years), higher household income is often associated with greater weight, whereas higher maternal education is associated with lower body mass index (BMI) [27]. By preschool age, interactions between income and maternal education become more pronounced: children in high-income but low-education households exhibit higher BMI, whereas this pattern reverses in households with more educated mothers. Center-based care attendance has been associated with healthier weight status [28]. Severe undernutrition is uncommon in this cohort, although slight growth deficits persist in lower-income groups [29]. Longer breastfeeding duration (up to 12 months) is linked with fewer socio-emotional difficulties in early childhood, whereas no benefits were observed for breastfeeding beyond 13 months [30].

### Cultural or ethnic variation

Indigenous (Mapuche) children exhibit slightly lower cognitive scores on average at ages 2–3, a difference largely attenuated when socioeconomic status is considered [31]. Some findings indicate that Mapuche children report fewer externalizing symptoms than non-Indigenous peers from similar backgrounds [31]. In supportive environments, cognitive progression among Indigenous children can match or exceed that of non-Indigenous peers [32]. Regional variation is also observed: children in rural areas score lower in language, but caregivers tend to report fewer behavioral difficulties compared to urban areas [11].

### Prenatal and early-life adversities

Children *in utero* during the February 2010 earthquake scored higher in emotional and behavioral symptoms in toddlerhood [33]. Self-reported maternal stress during pregnancy is negatively associated with cognitive scores [34]. Preterm birth correlates with lower cognitive and socio-emotional performance [35]. Postnatal contexts moderate these patterns: neighborhood insecurity is linked to weaker language development, but maternal self-efficacy has been associated with attenuating of such associations [36]. Exposure to domestic violence correlates with weaker socio-emotional functioning [37], while responsive parenting is associated with more favorable socio-emotional profiles [23]. One study compared toddlers assessed in 2021 with ELPI children measured in 2017 and found lower developmental scores post-pandemic, particularly in language, suggesting effects of disrupted caregiving and social experiences [38].

## What are the main strengths and weaknesses?

### Strengths

A core strength of ELPI lies in its longitudinal, nationally representative design, which captures children’s developmental trajectories across Chile’s diverse regions. Data collection occurs at multiple times and includes both standardized assessments and caregiver reports, allowing deeper analysis than single-time studies can provide. ELPI covers a wide range of domains—cognitive, socio-emotional, physical, and family context—enabling integrated research on how individual, household, and policy factors interact. Over successive waves, the study has been guided by different ministries, broadening its policy relevance and ensuring sustained national attention.

### Weaknesses

Despite robust designs, attrition remains a persistent challenge in all longitudinal studies, potentially biasing later findings unless addressed through appropriate weighting or imputation. In addition, there is limited consistency in the measurement of indicators across waves, not only due to the natural evolution of instruments—such as the inclusion of executive function assessments in 2017—but also as a result of institutional changes in survey administration. The ELPI was initially under the Ministry of Education (2010), then the Ministry of Labor and Social Welfare (2012), and later the Ministry of Social Development and Family (2017 and 2024). These shifts likely influenced priorities, instruments, and implementation strategies. Budget constraints in 2024 further prevented the inclusion of a new child cohort, focusing only on the 15 175 children from the original 2010 sample. Finally, while the study collects extensive information, the heavy reliance on self-reported data regarding behaviors and home environments may introduce reporting biases, despite standardized protocols.

## Can I get hold of the data? Where can I find out more?

Datasets from the ELPI waves can be accessed through the Banco Integrado de Datos (BIDAT), an official data repository managed by the Ministry of Social Development and Family. For more information or to access ELPI data and documentation (e.g. user manuals, codebooks, and de-identified databases), visit: https://bidat.gob.cl/directorio/Encuestas/elpi-cuarta-ronda-2024. For enquiries regarding data access, please contact: Gabriel Sotomayor (gsotomayor@desarrollosocial.gob.cl).

## Ethics approval

The preparation of this cohort profile did not require additional ethics approval because it is based on previously collected and publicly available de-identified data.

## Data Availability

No new data were generated for this article. ELPI datasets are available through the BIDAT repository, as described in the “Can I get hold of the data?” section.
